# Measuring multimorbidity in hospitalised patients using linked hospital episode data: comparison of two measures

**DOI:** 10.23889/ijpds.v4i1.461

**Published:** 2019-01-21

**Authors:** Lynn Robertson, Dolapo Ayansina, Marjorie Johnston, Angharad Marks, Corri Black

**Affiliations:** 1 Aberdeen Centre for Health Data Science, University of Aberdeen, Aberdeen, Scotland; 2 Institute of Applied Health Sciences, University of Aberdeen, Aberdeen, Scotland; 3 Renal Department, NHS Grampian, Aberdeen, Scotland; 4 The Farr Institute of Health Informatics Research, University of Aberdeen, Aberdeen, Scotland; 5 Public Health Directorate, NHS Grampian, Aberdeen, Scotland

## Abstract

**Introduction:**

Multimorbidity is a complex and growing health challenge. There is no accepted “gold standard” multimorbidity measure for hospital resource planning, and few studies have compared measures in hospitalised patients.

**Aim:**

To evaluate operationalisation of two multimorbidity measures in routine hospital episode data in NHS Grampian, Scotland.

**Methods:**

Linked hospital episode data (Scottish Morbidity Record (SMR)) for the years 2009-2016 were used. Adults admitted to hospital as a general/acute inpatient during 2014 were included. Conditions (ICD-10) were identified from general/acute (SMR01) and psychiatric (SMR04) admissions during the five years prior to first admission in 2014. Two count-based multimorbidity measures were used (Charlson Comorbidity Index and Tonelli et al.), and multimorbidity was defined as ≥2 conditions. Kappa statistics assessed agreement. The association between multimorbidity and length of stay, readmission and mortality was assessed using logistic and negative binomial regression as appropriate.

**Results:**

In 41,545 adults (median age 62 years, 52.6% female), multimorbidity prevalence was 15.1% (95% CI 14.8%, 15.5%) using Charlson and 27.4% (27.0%, 27.8%) using Tonelli – agreement 85.1% (Kappa 0.57). Multimorbidity prevalence, using both measures, increased with age. Multimorbidity was higher in males (16.5%) than females (13.9%) using the Charlson measure, but similar across genders when measured with Tonelli. After adjusting for covariates, multimorbidity remained associated with longer length of stay (Charlson IRR 1.1 (1.0, 1.2); Tonelli IRR 1.1 (1.0, 1.2)) and readmission (Charlson OR 2.1 (1.9, 2.2); Tonelli OR 2.1 (2.0, 2.2)). Multimorbidity had a stronger association with mortality when measured using Charlson (OR 2.7 (2.5, 2.9)), than using Tonelli (OR 1.8 (1.7, 2.0)).

**Conclusions:**

Multimorbidity measures operationalised in hospital episode data identified those at risk of poor outcomes and such operationalised tools will be useful for future multimorbidity research and use in secondary care data systems. Multimorbidity measures are not interchangeable, and the choice of measure should depend on the purpose.

**Highlights:**

- Operationalisation of two count-based multimorbidity measures using linked electronic hospital episode data was evaluated (Charlson and Tonelli).
- First study to compare the Tonelli measure with another measure for investigating multimorbidity in hospitalised patients.
- Multimorbidity prevalence differed depending on measure used, but both multimorbidity measures identified those at risk of poor outcomes.
- Operationalised multimorbidity tools have uses for future multimorbidity research and use in secondary care data systems.
- Multimorbidity measures are not interchangeable, and choice of measure should depend on purpose.

## Introduction

Multimorbidity is the coexistence of multiple conditions, usually defined as two or more, in the same individual[1]. Alongside an ageing population, multimorbidity is a growing public health concern and a key research priority at an international policy level[2-5]. Multimorbidity is associated with poor health outcomes, high health service utilisation, and high health care costs[6-10].

In addition to the importance of multimorbidity/comorbidity measures in risk adjustment to reduce confounding, operational multimorbidity measures/tools are vital for several reasons. In hospitalised populations, estimates of the prevalence of multimorbidity are necessary to assess the impact of multimorbidity on resources. Multimorbidity tools are needed to identify patients at risk of poor outcomes at point of admission, thus enabling more effective care, discharge planning and improved outcomes. Identifying individuals with multimorbidity has been highlighted as important in international goals, policy[2-4] and national guidelines[11].

The prevalence of multimorbidity in hospitalised patients varies widely among studies (22% to 99.7%)[12-17]. Some of this difference is likely to be due to different methods used for measuring multimorbidity including how multimorbidity is defined, the number and type of conditions included, data sources and coding schemes, population studied, and setting[18]. Multimorbidity is complex to measure and currently there is no universal “gold standard”[1]. Numerous heterogeneous measures exist[1,18-20], falling into two broad types. First, weighted indices, of which the most commonly used is the Charlson Comorbidity Index[21]. Second, and more commonly used in multimorbidity studies[19], simple counts of conditions or diagnostic categories, which utilize a selected list of conditions or diagnostic categories, or all conditions within a population. More recently, a simple count of the Charlson Comorbidity Index conditions has been used in multimorbidity research[15,22,23]. A count-based multimorbidity measure published in a “landmark” study by Barnett et al.[24], identified 40 chronic conditions for measuring multimorbidity in a primary care population from clinical coding and prescribing data, using data coding unique to the UK (Read codes). This study has been widely cited and many studies have reported using this measure[25-28]. Subsequently, a corresponding coding scheme for use with administrative data based on the International Classification of Diseases (ICD) system was developed by Tonelli et al.[29].

Several studies have directly compared weighted multimorbidity measures or compared weighted with simple count measures[30-36]. Studies have also directly compared simple count-based multimorbidity measures in primary care and general populations, reporting prevalence, emergency admission, functional decline, and physical quality of life[22,37,38]. We are aware of only two studies, however, that have directly compared any simple count-based measures in hospitalised patients[15,23]. Dattalo et al.[23] compared four count-based measures based on ICD coding, but focussed on patients aged 65 years and older. Prevalence estimates varied depending on measure, and there was an increased risk of 30-day readmission for patients with multimorbidity for each of the four measures. Schneider et al.[15] compared three count-based measures in a small study of medical inpatients aged 18 years and over admitted from the emergency unit reporting that prevalence estimates varied for the three measures. Although multimorbidity measures have been extensively studied, relatively little is known about count-based measures in general hospitalised populations, particularly in younger patients. To our knowledge, the Tonelli coding scheme has not been studied in a hospitalised population for assessing multimorbidity prevalence or outcomes.

The aim of this study was to operationalise and apply two count based measures of multimorbidity (Charlson[39] and Tonelli[29]) to routine hospital episode data, in order to a) compare prevalence; b) assess agreement, and c) compare the association between multimorbidity and length of hospital stay, hospital readmission and mortality.

## Methods

### Study design and setting

**Figure 1: Overview of study design:**
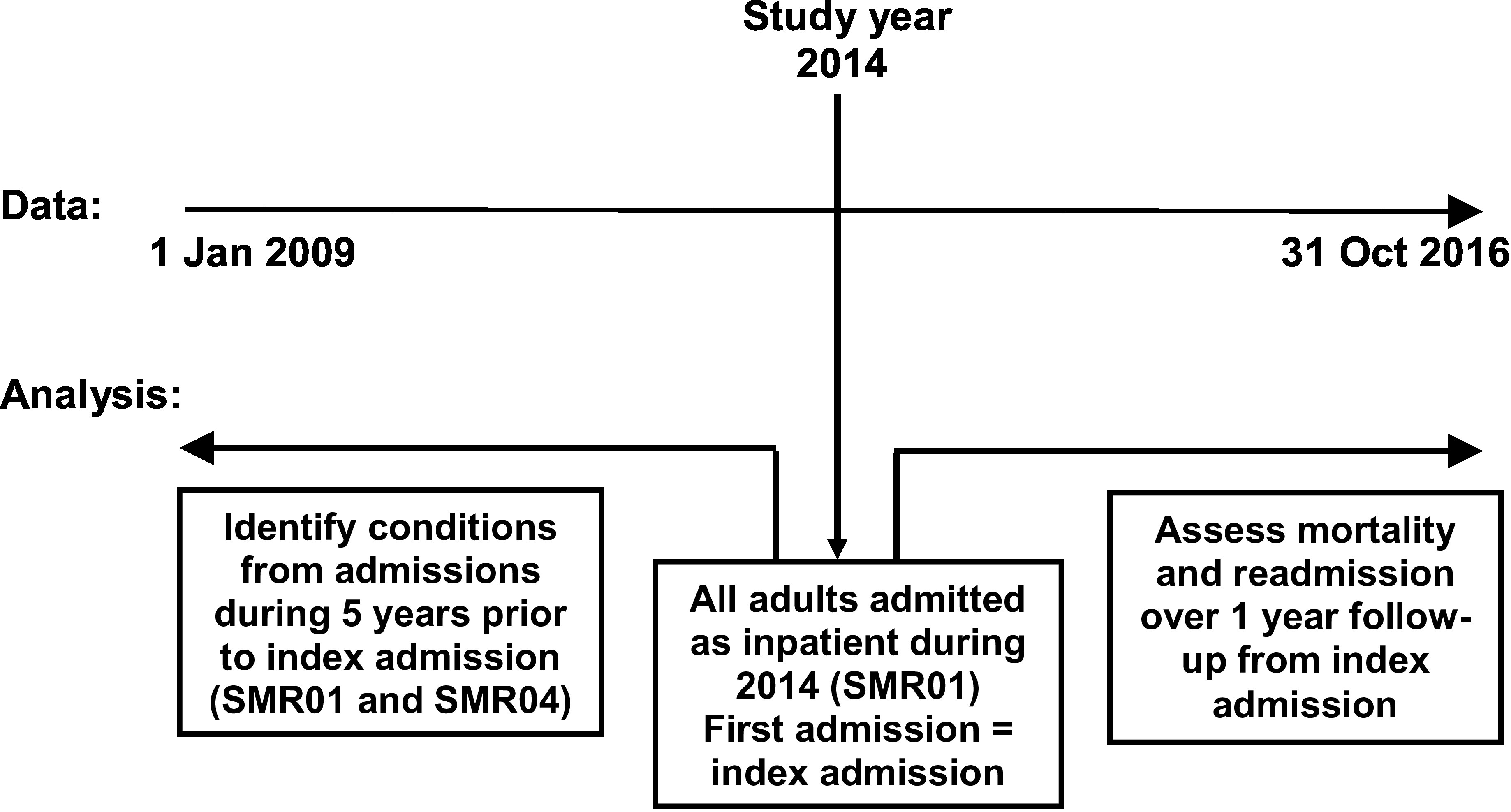
SMR, Scottish Morbidity Record (SMR01 general/acute, SMR04 psychiatric)

This study is reported as per RECORD guidelines[40]. This was a population-based observational study using linked electronic health records carried out in a secondary care setting in a single health region in the north-east of Scotland (Grampian region, total population 2014, 584,220[41]). An overview of the study design is shown in Figure 1.

#### Data sources

We used hospital episode data, Scottish Morbidity Record (SMR)[42], from general/acute (SMR01) and psychiatric (SMR04) admissions, from the years 2009-2016. SMR is an episode-based patient record relating to all patients discharged from hospital in Scotland. A record is generated when a patient completes an episode of care (period of time spent under the care of one consultant). These episodes are then linked to form a continuous inpatient stay (CIS) representing one admission, which may include transfers between consultants, specialties and/or hospitals. SMR data is collated in a national database, managed by Information Services Division Scotland (ISD)[43], and data is returned to each regional health authority on an ongoing basis. Data collected include patient identifiable and demographic details, episode management details, general clinical information and death data. Clinical information is recorded as main diagnosis and up to five other significant diagnoses, and coded using ICD-10. Deprivation information was collected from the Scottish Index of Multiple Deprivation (SIMD)[44], and rurality from the Scottish Government Urban Rural Classification system[45].

#### Study population

**Figure 2: Flowchart of study population and data linkage:**
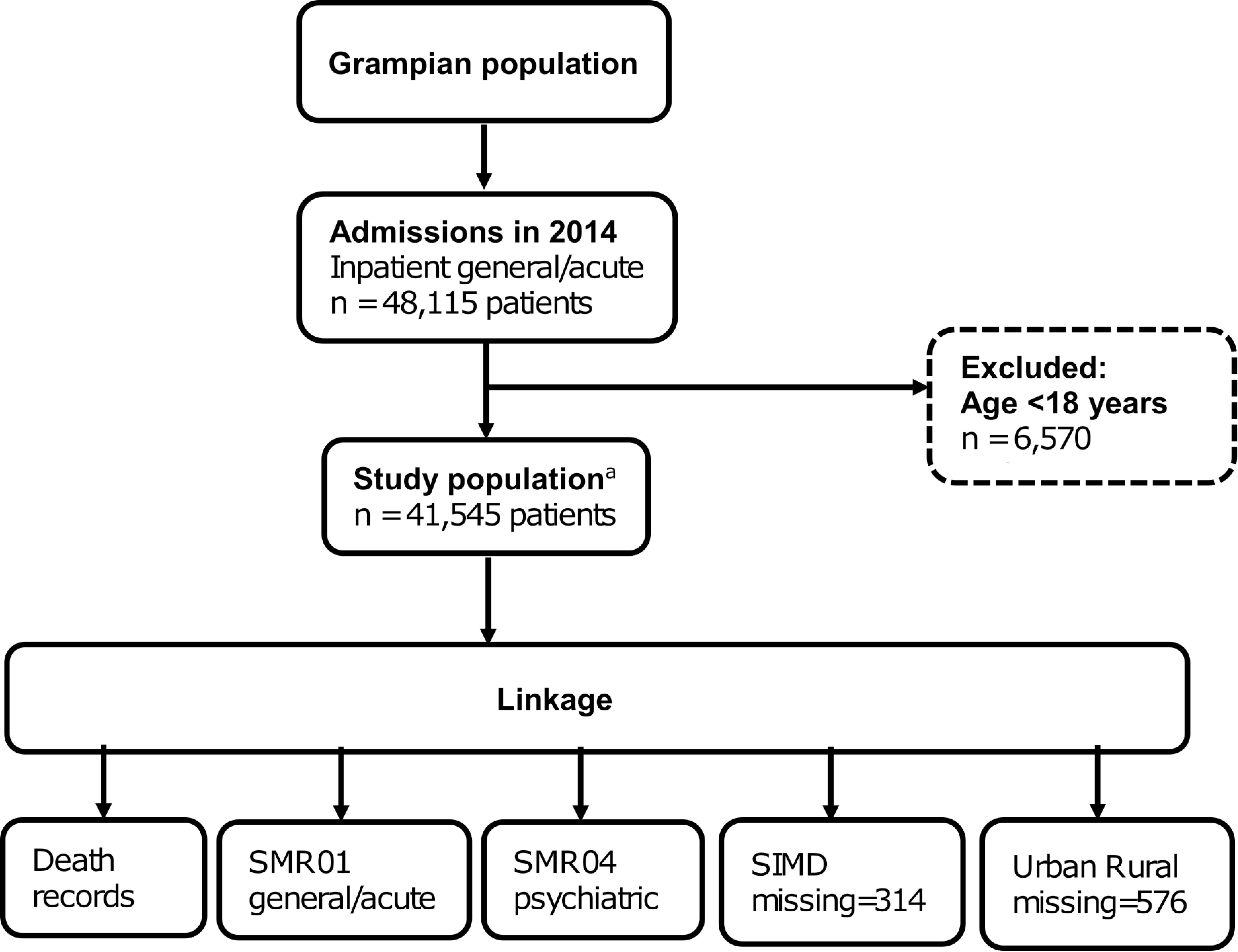
SMR, Scottish Morbidity Record; SIMD, Scottish Index of Multiple Deprivation^a^ CHI number was missing or invalid for 662 inpatient general/acute admissions in 2014 (patients ≥18 years), therefore not included in the study population.

We included all adult patients (≥18 years) admitted to hospital as an inpatient during 2014 (SMR01 only), for a single regional health authority (NHS Grampian). We excluded day case, obstetric and psychiatric admissions when identifying the index admission. A patient’s first admission in 2014 was classified as their “index admission”, and the admission date was classified as their “index date”. The flow diagram for identifying the study population is shown in Figure 2. From index date in 2014, follow-up data were available to 31 October 2016.

### Multimorbidity

Multimorbidity was defined *a priori* as ≥2 conditions[1,11], measured using an unweighted simple count of conditions. Conditions were identified from general/acute (SMR01, including day cases) and psychiatric (SMR04) admissions in the five years prior to index date, recorded as either main or other diagnosis. We compared two measures. The first was a count of the 17 conditions included in the Charlson Comorbidity Index, based on ICD-10 coding[39] (Charlson measure). The second measure was a count of the 30 chronic conditions developed by Tonelli et al.[29], based on ICD-10 coding (Tonelli measure). For each measure, multiple pre-specified ICD-10 codes were used to define each condition. Twelve conditions were common to both the Charlson and Tonelli measures. However, only dementia, rheumatic disease and metastatic cancer had identical ICD-10 codes for both measures. While the Charlson measure included hemiplegia or paraplegia and AIDS, these conditions were not included in the Tonelli measure. The Tonelli measure on the other hand, included many conditions not included in the Charlson measure: hypertension, alcohol misuse, atrial fibrillation and flutter, chronic pain, depression, epilepsy, hypothyroidism, inflammatory bowel disease, irritable bowel syndrome, multiple sclerosis, Parkinson’s disease, psoriasis, schizophrenia, and severe constipation. The specific ICD-10 codes for conditions included in each measure are detailed in Supplementary Appendix 1, with a note of minor amendments made to the Tonelli measure. These codes were translated into computerised algorithms and applied to SMR data to identify the conditions of interest. For data quality purposes, a validation dataset containing all ICD-10 codes for main and other diagnoses recorded in the five years prior to index date for a random sample of 50 patients was manually checked against the final dataset. This showed that the computerized algorithms correctly captured conditions for all patients in the sample.

### Other covariates

Other baseline characteristics were sex, age, deprivation, rurality, and admission type (routine or emergency). Age was categorised into six age groups. Deprivation was measured using SIMD quintiles[44], and rurality was measured using the Scottish Government 6 fold Urban Rural Classification[45].

### Outcomes

Study outcomes were multimorbidity prevalence, length of stay of index admission, calculated as the number of days from admission date until discharge date of the whole CIS (discharged alive or dead), hospital readmission (whole CIS, including general/acute inpatient and day case admissions, excluding psychiatric and obstetric admissions) up to one year from discharge date and all-cause mortality up to one year from index date.

### Data linkage

NHS Grampian SMR data were held in a dedicated secure server, managed by the accredited Grampian Data Safe Haven (DaSH)[46]. The Community Health Index (CHI) number, a unique patient identifier used throughout the Scottish health care system, was used to link the study population to hospital episode and death data, using deterministic matching. Postcodes were used to link the study population to SIMD and Urban Rural Classification. The de-identified dataset was prepared and hosted by the Grampian DaSH[46], allowing secure controlled access for researchers while ensuring data security.

There were 662 admissions in 2014 (inpatient general/acute, ≥18 years) with missing CHI numbers, therefore these were not included in our study population. There were 314 patients who could not be linked with SIMD, and 576 patients who could not be linked with Urban Rural Classification, because of postcode issues (Figure 2). The characteristics of patients with missing values are reported in Supplementary Appendix 2.

### Statistical analysis

Baseline characteristics were described as frequencies and percentages or as median and interquartile range (IQR). We calculated the prevalence of multimorbidity, and 95% confidence intervals (CI), as the proportion of patients with ≥2 conditions. Prevalence was reported by age group, sex, admission type (routine or emergency), SIMD quintile and Urban Rural category. Agreement between the two measures for classifying patients as multimorbid was assessed using the Kappa-statistic, by sex and age group (<75/≥75 years). We categorised Kappa scores as follows: κ ≤ 0.20 = poor, 0.21 ≤ κ ≤ 0.40 = fair, 0.41 ≤ κ ≤ 0.60 = moderate, 0.61 ≤ κ ≤ 0.80 = substantial, κ > 0.80 = good. To assess the association between variables, we used a χ^2^ test, Wilcoxon rank sum and Kruskal-Wallis test as appropriate. A complete case analysis of the association between multimorbidity and length of stay was assessed using multivariate negative binomial regression to estimate unadjusted and adjusted incident rate ratios (IRR) with 95% CI. The association between multimorbidity and readmission within one year of discharge date and mortality within one year of index date was assessed using multivariate logistic regression to estimate unadjusted and adjusted odds ratios (OR). All models were adjusted for age, sex, admission type, SIMD quintile and Urban Rural category. To assess model fit for mortality and readmission, we computed pseudo R2 (Cox & Snell/Nagelkerke). For length of stay we computed the Akaike information criterion (AIC) and the Bayesian information criterion (BIC). Analyses were performed using Stata v13.0 and SPSS v24.

### Ethical approval

The study was approved by the North of Scotland Research Ethics Service (REC B Ref. 16/NI/0088), NHS Grampian Research and Development (Ref. 2016UA006) and NHS Grampian Caldicott Guardian.

## Results

### Baseline characteristics

There were 41,545 patients included with a median age of 62 years (IQR 44-75 years) and 52.6% were female (Table 1). The majority of patients were admitted as an emergency (69.3%). Just over half of patients were from the two least deprived quintiles (52.4%), and urban categories (52.7%); comparable with the population distribution in the Grampian region. Approximately two-thirds (64.5%) of patients had been admitted to hospital at least once in the five years prior to index date.

**Table 1: Baseline characteristics and prevalence of multimorbidity by two measures:** CI, confidence interval; SIMD, Scottish Index of Multiple Deprivation; IQR, interquartile range. ^a^ 314 patients had missing values for SIMD category and 576 patients had missing values for Urban Rural category.

	Total		Charlson ≥2 conditions		Tonelli ≥2 conditions	
			
	n	(%)	%	(95% CI)	%	(95% CI)

**All patients**	41,545		15.1%	(14.8-15.5)	27.4%	(27.0-27.8)
**Sex**						
Male	19,677	(47.4%)	16.5%	(16.0-17.0)	27.1%	(26.4-27.7)
Female	21,868	(52.6%)	13.9%	(13.5-14.4)	27.7%	(27.1-28.3)
**Age groups**						
18-29	4,677	(11.3%)	1.1%	(0.8-1.4)	5.9%	(5.3-6.6)
30-44	5,932	(14.3%)	3.7%	(3.2-4.2)	11.0%	(10.3-11.9)
45-59	8,671	(20.9%)	8.4%	(7.8-9.0)	19.0%	(18.2-19.8)
60-74	11,160	(26.9%)	19.3%	(18.6-20.1)	32.8%	(31.9-33.7)
75-89	9,705	(23.4%)	28.3%	(27.4-29.2)	45.8%	(44.8-46.8)
≥90	1,400	(3.4%)	28.0%	(25.7-30.4)	50.1%	(47.5-52.8)
**Admission type**						
Routine	12,754	(30.7%)	11.3%	(10.7-11.8)	22.6%	(21.9-23.4)
Emergency	28,791	(69.3%)	16.9%	(16.4-17.3)	29.5%	(29.0-30.1)
**SIMD 2012^a^**						
1 (most deprived)	3,317	(8.0%)	15.2%	(14.0-16.5)	28.7%	(27.2-30.3)
2	6,279	(15.1%)	16.3%	(15.4-17.2)	29.2%	(28.1-30.4)
3	9,886	(23.8%)	15.8%	(15.1-16.5)	28.7%	(27.8-29.6)
4	10,792	(26.0%)	15.0%	(14.3-15.7)	26.6%	(25.8-27.5)
5 (least deprived)	10,957	(26.4%)	14.3%	(13.6-14.9)	26.0%	(25.2-26.9)
**Urban Rural^a^**						
Large urban	15,577	(37.5%)	15.8%	(15.2-16.3)	28.8%	(28.1-29.5)
Other urban	6,329	(15.2%)	16.1%	(15.2-17.0)	29.1%	(28.0-30.2)
Accessible small town	3,329	(8.0%)	16.6%	(15.3-17.9)	27.8%	(26.3-29.3)
Remote small town	3,719	(9.0%)	15.5%	(14.4-16.7)	28.3%	(26.9-29.8)
Accessible rural	7,484	(18.0%)	14.7%	(13.9-15.5)	26.7%	(25.7-27.7)
Remote rural	4,531	(10.9%)	12.0%	(11.1-13.0)	22.0%	(20.9-23.3)
**Admitted in previous 5 years**						
Yes	26,780	(64.5%)				
No	14,765	(35.5%)				
**Number of Charlson conditions**						
0	27,560	(66.3%)	-	-	-	-
1	7,697	(18.5%)	-	-	-	-
2	3,867	(9.3%)	-	-	-	-
3	1,578	(3.8%)	-	-	-	-
4	578	(1.4%)	-	-	-	-
5	195	(0.5%)	-	-	-	-
6	61	(0.1%)	-	-	-	-
7	9	(0.0%)	-	-	-	-
**Number of Tonelli conditions**						
0	22,884	(55.1%)	-	-	-	-
1	7,272	(17.5%)	-	-	-	-
2	5,173	(12.5%)	-	-	-	-
3	3,241	(7.8%)	-	-	-	-
4	1,665	(4.0%)	-	-	-	-
5	783	(1.9%)	-	-	-	-
6	357	(0.9%)	-	-	-	-
7	100	(0.2%)	-	-	-	-
8	56	(0.1%)	-	-	-	-
9	9	(0.0%)	-	-	-	-
10+	5	(0.0%)	-	-	-	-
**Length of stay, days (median, IQR)**	2	(1-6)	3	(1-8)	3	(1-8)
**Readmission within 1 year of discharge, n (%)**	18,318	(44.1%)	3,976	(63.2%)	6,861	(60.2%)
**Died within 1 year of index date, n (%)**	4,619	(11.1%)	1,860	(29.6%)	2,431	(21.3%)

### Prevalence of individual conditions

Of the individual conditions recorded in the five years prior to index admission that contributed to the Charlson measure, those with the highest prevalence were chronic pulmonary disease (11.3%), diabetes without complications (7.5%), malignancy (6.9%), renal disease (6.1%) and myocardial infarction (5.6%). The individual conditions with the highest prevalence from the Tonelli measure were hypertension (19.0%), diabetes (8.4%), chronic kidney disease (8.2%), asthma (6.7%) and atrial fibrillation and flutter (6.1%) (Supplementary Appendix 1).

### Prevalence of multimorbidity

Counts of conditions ranged from 0-7 for Charlson and 0-11 for Tonelli (Table 1). Sixty-six percent of the population had 0 conditions using Charlson, compared to 55.1% when using Tonelli (Table 1). The proportion of patients with multimorbidity (≥2 conditions) for each measure is presented in Table 1. Overall, the prevalence of multimorbidity was lower when measured using Charlson compared to Tonelli (overall 15.1% vs 27.4% respectively).

**Figure 3: Prevalence of multimorbidity by age and sex:**
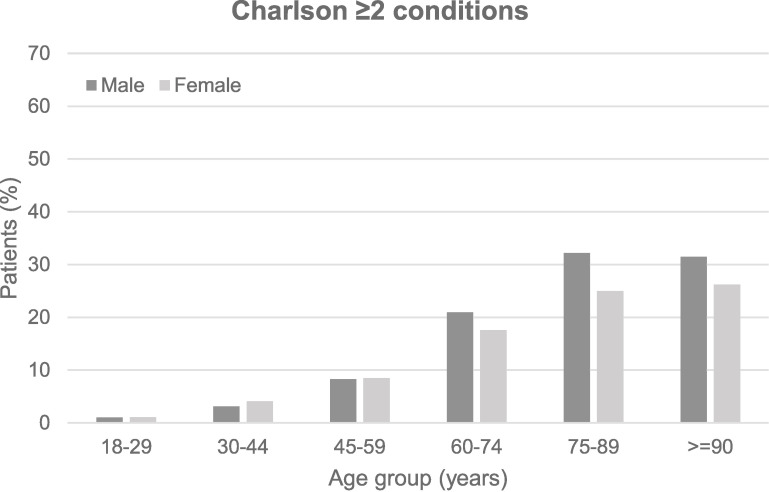

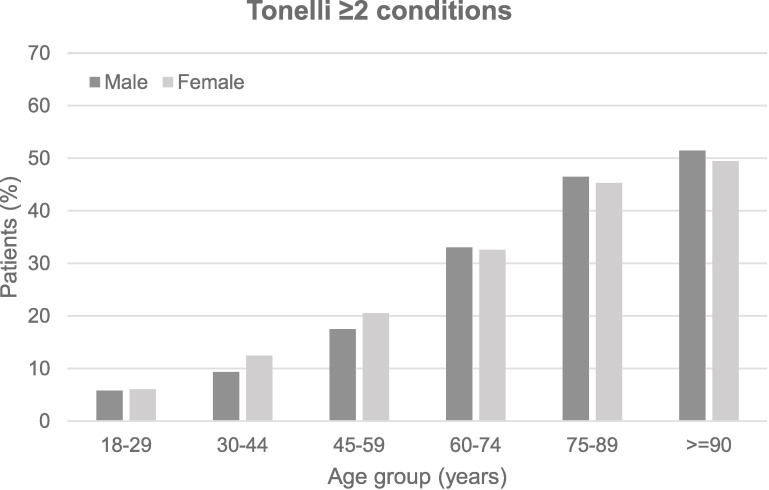


The proportion of patients with multimorbidity increased with age (Table 1). Figure 3 shows the prevalence of multimorbidity by gender, for different age groups. At younger ages (<60 years), females had similar or very slightly higher prevalence of multimorbidity than males using both measures, whereas at older ages (≥60 years), this seemed to reverse and males had a slightly higher prevalence of multimorbidity than females. However, overall, the prevalence of multimorbidity was higher in males than females when measured using Charlson (16.5% and 13.9% respectively; p<0.001), but similar for males and females when measured using Tonelli (27.1% and 27.7% respectively) (Table 1).

**Percent with ≥2 Charlson conditions:** 

	18-29	30-44	45-59	60-74*	75-89*	≥90*

Male	1.0%	3.2%	8.3%	21.0%	32.2%	31.5%
Female	1.1%	4.1%	8.5%	17.6%	25.0%	26.2%

**Percent with ≥2 Tonelli conditions:** ^*^ difference between genders by age group p<0.05.

	18-29	30-44*	45-59*	60-74	75-89	≥90

Male	5.8%	9.3%	17.5%	33.0%	46.5%	51.5%
Female	6.0%	12.5%	20.5%	32.6%	45.3%	49.5%

### Agreement between multimorbidity measures in classifying patients as multimorbid

Overall, 85.1% of the study population was consistently classified by Charlson and Tonelli as either multimorbid or not, showing moderate agreement between the two measures (Kappa 0.57) (Table 2). Agreement was slightly higher for males (agreement 86.1%, Kappa 0.60) compared to females (agreement 84.3%, Kappa 0.54). Agreement was higher for patients <75 years (agreement 87.8%, Kappa 0.54) compared to ≥75 years (agreement 77.9%, Kappa 0.54).

**Table 2: Agreement between Tonelli and Charlson:** 

Group	Total	Agreement				Discordant				Agreement	Kappa
			
		Charlson <2		Charlson ≥2		Charlson <2		Charlson ≥2			
		Tonelli <2		Tonelli ≥2		Tonelli ≥2		Tonelli <2		%	
		n	%	n	%	n	%	n	%		

**Total**	41,545	29,620	(71.3)	5,752	(13.8)	5,637	(13.6)	536	(1.3)	85.14	0.57
**Sex**											
Males	19,677	14,024	(71.3)	2,915	(14.8)	2,408	(12.2)	330	(1.7)	86.09	0.60
Females	21,868	15,596	(71.3)	2,837	(13.0)	3,229	(14.8)	206	(0.9)	84.29	0.54
**Age group**											
Age <75	30,440	23,884	(78.5)	2,838	(9.3)	3,403	(11.2)	315	(1.0)	87.79	0.54
Age ≥75	11,105	5,736	(51.6)	2,914	(26.2)	2,234	(20.1)	221	(2.0)	77.89	0.54

### Outcomes

All patients had been discharged (alive or dead) by the end of follow-up (31 October 2016). The overall median length of stay was 2 days (IQR 1-6). Length of stay was longer for patients with multimorbidity compared to those with <2 conditions (Charlson adjusted IRR 1.1 (95% CI 1.0, 1.2); Tonelli adjusted IRR 1.1 (95% CI 1.0, 1.2)) (Table 3).

**Table 3: Association between multimorbidity and length of stay, readmission and mortality:** IQR, interquartile range; IRR, incident rate ratio; OR, odds ratio; CI, confidence interval. ^a^ 9 patients did not have a full year of follow-up data from discharge date to 31 October 2016, of whom 2 were readmitted, and 7 were not readmitted up to 31 October 2016. ^b^ 1,403 patients died during index admission and 3,216 died following index admission but within 1 year of index date. ^*^ p=0.005 ^**^ p<0.001 ^***^ p=0.001 Notes: All models adjusted for age, sex, type of admission (routine or emergency), SIMD quintile and Urban Rural category. 621 patients had missing values for SIMD and/or Urban Rural category, therefore not included in the adjusted models. Model fit for Length of Stay: Charlson AIC 230767, BIC 230896; Tonelli AIC 230748, BIC 230877 Model fit for Readmission: Charlson Cox & Snell 0.039, Nagelkerke 0.053; Tonelli Cox & Snell 0.048, Nagelkerke 0.064 Model fit for Mortality: Charlson Cox & Snell 0.140, Nagelkerke 0.278; Tonelli Cox & Snell 0.132, Nagelkerke 0.261

	Total	Length of stay		Readmitted 1 year from discharge date^a^		Died 1 year from index date^b^	
	
	n	median (IQR)	adjusted IRR (95% CI)	Number readmitted	adjusted OR (95% CI)	Number died	adjusted OR (95% CI)

**Charlson**							
<2 conditions	35,257	2 (1-5)	Reference	14,342	Reference	2,759	Reference
≥2 conditions	6,288	3 (1-8)	1.1 (1.0, 1.2)*	3,976	2.1 (1.9, 2.2)**	1,860	2.7 (2.5, 2.9)**
**Tonelli**							
<2 conditions	30,156	2 (1-5)	Reference	11,457	Reference	2,188	Reference
≥2 conditions	11,389	3 (1-8)	1.1 (1.0, 1.2)***	6,861	2.1 (2.0, 2.2)**	2,431	1.8 (1.7, 2.0)**

A total of 18,318 patients (45.6% of patients discharged alive) were readmitted at least once within one year of discharge date. Patients with multimorbidity had a higher risk of readmission compared to those with <2 conditions (Charlson adjusted OR 2.1 (95% CI 1.9, 2.2); Tonelli adjusted OR 2.1 (95% CI 2.0, 2.2) (Table 3).

A total of 4,619 (11.1%) patients died within one year of index date. Of these 1,403 died during index admission and 3,216 died following index admission but within one year of index date. Patients with multimorbidity had a higher risk of death compared to those with <2 conditions (Charlson adjusted OR 2.7 (95% CI 2.5, 2.9); Tonelli adjusted OR (95% CI 1.8 (1.7, 2.0)) (Table 3).

## Discussion

In our study of hospital patients, we found that the prevalence of multimorbidity was higher when measured using Tonelli, compared with Charlson. Both measures found that multimorbidity increased with age, but there were differences between the measures for gender, with a higher prevalence of multimorbidity in males than females using Charlson, but similar for males and females when using Tonelli. There was moderate agreement between the two measures in classifying patients as multimorbid or not, with more agreement evident in males and younger patients. Multimorbidity, measured using both Tonelli and Charlson, was associated with an increased risk of longer length of stay, readmission and mortality. To our knowledge, this is the first study to compare the Tonelli measure with another multimorbidity measure, in a hospitalised adult population.

We found moderate agreement between Tonelli and Charlson when classifying patients as multimorbid, and that the overall prevalence of multimorbidity was higher when measured using Tonelli (27.4%) compared to Charlson (15.1%). Our finding of a higher prevalence using Tonelli, which includes a larger number of conditions than Charlson, was not unexpected, and is consistent with other studies in primary care[18,22,37,38] and hospitalised patients[15].

Although several studies have compared weighted measures[33-36], to our knowledge only two previous studies compared count-based multimorbidity measures in hospitalised patients[15,23]. Neither of these studies used the Tonelli measure (based on the landmark Barnett study[24]), although both included a count of Charlson conditions. Dattalo et al.[23] compared four count-based measures in patients aged 65 and older, based on ICD coding. Comparing Charlson to the other three measures, they reported Kappa values of 0.07, 0.36 and 0.44 – lower agreement than we found when comparing Charlson with Tonelli (Kappa 0.57). They also reported a wide variation in the prevalence of multimorbidity between measures (18.6% to 92.9%). Prevalence did not seem to increase with the number of conditions included, but there were methodological differences in how the measures were operationalised which may explain this. Using the Charlson measure, Dattalo et al.[23] reported a multimorbidity prevalence of 36.8%, which was higher than we found in patients of a similar age (≥60 years, 23.8%). However, we used only inpatient admission data, whereas Dattalo et al.[23] included inpatient and outpatient data to identify Charlson conditions[23]. The other study by Schneider et al.[15] compared three count-based measures, reporting a multimorbidity prevalence of 48.2% for the Charlson measure, considerably higher than we have reported. However, this was a small study of medical inpatients admitted from the emergency unit, which may represent a patient population with a high severity of illness[15].

We found that the prevalence of multimorbidity increased with age, for both measures, which is a well-recognised association. Although multimorbidity increased with age, we still found substantial multimorbidity in younger age groups, particularly using Tonelli, as shown in Table 1. The prevalence of multimorbidity was higher in males than females when measured using Charlson, but similar for males and females when using Tonelli. This finding is not consistent with previous reports in primary care populations, where multimorbidity is generally more common in females[6,12,24]. Studies reporting the prevalence of multimorbidity by gender in hospitalised populations are limited, and report conflicting results[14,16,47,48]. We also noted some evidence of a different pattern of multimorbidity between males and females at different age groups. Any age and sex differences are likely to be a reflection of the specific conditions included in the two measures. The majority of conditions included in the Charlson measure may be more prevalent in older individuals and males, whereas the Tonelli measure includes conditions that may be more common in younger individuals (for example, asthma, epilepsy, psoriasis), and females (for example, chronic pain, depression, hypothyroidism).

Multimorbidity, measured using both Charlson and Tonelli, was associated with an increased risk of longer length of stay, readmission, and mortality. Length of stay and readmission risk was similar for both measures. There was a stronger association between multimorbidity and mortality when measured using Charlson, compared with Tonelli, but the model fit was similar. This finding was not unexpected given that Charlson was developed to predict mortality[21], and includes many drivers of all-cause mortality, whereas there were more in number but relatively less mortal conditions included in Tonelli. Dattalo et al.[23] reported an increased risk of 30-day readmission for patients aged 65 and older with multimorbidity, for each of the four measures they assessed, reporting ORs ranging from 1.1 to 1.5, and that Charlson and Medicare Advantage Chronic Condition Special Needs Plan were the best predictors of 30-day readmission.

This was a large, population-based study, not limited to an age or patient group. We ascertained conditions over the five years prior to index date, as longer lookback periods have been shown to be better at identifying patients with chronic conditions[49-51]. We used high quality administrative data[52], and undertook quality assurance assessments to ensure accuracy of coding algorithms. We used two high profile measures: Tonelli et al.[29], based on the landmark Barnett et al. study[24], as well as the widely used Charlson measure[39]. Furthermore, to our knowledge, this is the first study to use the Tonelli measure for investigating multimorbidity in hospitalised patients, and first to compare Tonelli with another measure.

Limitations, however, should be recognised. Conditions were identified from hospital episode data in the five years prior to admission in 2014, and 35.5% of our population had no admission in the previous five years. As a result, we will not have recorded conditions for patients who were first time presenters, and will have underestimated the multimorbidity burden in our population, especially for conditions that do not lead to hospitalisation or which are not a priority for recording on discharge records. This situation is likely to have more effect on the Tonelli conditions than the Charlson conditions. However, as hospital episode data may be the only information available to clinicians when a patient is admitted, we feel that using this methodology is relevant and important to examine. This also highlights the importance of an integrated primary and secondary care patient record, which would provide more information to clinicians and a fuller picture of multimorbidity. We could not explore differences in multimorbidity by ethnicity, as the Grampian population is ethnically homogeneous with only ~4% of the population non-White ethnic minorities[53]. We have used simple counts of conditions and as such have applied uniform weights to all conditions and therefore take no account of severity of illness. Weighted measures should be used where they are validated for a particular outcome of interest. However, where there are multiple outcomes being considered in a study, the use of disease counts is more appropriate[19,24,54]. Thus, the approach in our study is reasonable but we also acknowledge that our conclusions are only valid in the context of using simple count-based measures. Finally, we compared only two measures of multimorbidity, acknowledging that many measures exist. Both measures were selected on the basis of their high profile nature. Charlson was included as it is a widely adopted validated measure[19]. Tonelli was included as it was an adaptation of the landmark study by Barnett et al.[24] Tonelli addressed the requirement for ICD coding algorithms for the Barnett conditions.

It should also be highlighted, that the study by Tonelli et al.[29] did not identify appropriate algorithms for all 40 conditions included in the Barnett et al.[24] measure. There were also some differences in the specific conditions included in the measure (Supplementary Appendix 3). In order to fully implement the Barnett measure, ICD coding algorithms for all the conditions would be valuable.

The methodology used in our study with regard to either measure of multimorbidity would be applicable to health systems worldwide that use the ICD-10 coding system. The findings for the prevalence and outcomes of multimorbidity would likely apply to other hospitalised populations with similar characteristics to our study population.

Information on the prevalence and outcomes of multimorbidity in hospitalised patients is essential for service planning, clinical decision-making and clinical research. At point of admission, assessment may benefit from the inclusion of a multimorbidity tool to identify high risk patients, and to plan patient care. Our findings have implications for the choice of measure. For investigating the burden of multimorbidity, it may be more useful to use a more comprehensive measure which includes a large number of conditions, such as Barnett et al.[24] or Tonelli et al.[29], facilitating comparison with other studies using similar measures. For identifying high risk patients, it makes little difference which measure is used, at least for the outcomes investigated in our study. Charlson, however, is less data intensive and more strongly associated with mortality. We have demonstrated the potential of linking electronic health records to identify hospitalised patients at risk of worse outcomes for assessment and targeted intervention. An integrated primary and secondary care patient record would provide more information to clinicians at point of admission.

## Conclusion

Multimorbidity measures operationalised in electronic hospital episode data identified those at risk of poor outcomes and such operationalised tools will be useful for future multimorbidity research and use in secondary care data systems, for example to assess multimorbidity at point of admission in order to inform patient management and ongoing care. Multimorbidity measures are not interchangeable, and choice of measure will depend on purpose.

## Data sharing statement

De-identified data used for this study are held by Grampian Data Safe Haven. These data are available provided the necessary permissions have been obtained. Further information is available at http://www.abdn.ac.uk/iahs/facilities/grampian-data-safe-haven.php and requests for data may be made to Professor Corri Black on behalf of Grampian Data Safe Haven, corri.black@abdn.ac.uk.

## Supplementary Appendices

Supplementary Appendix 1. Coding definitions (ICD-10) for conditions included in Charlson and Tonelli measures.

Supplementary Appendix 2. Characteristics of patients with missing CHI numbers, SIMD quintile or UR category.

Supplementary Appendix 3. Tonelli et al. measure observations.

## Abbreviations

AIC	Akaike information criterion
BIC	Bayesian information criterion
CHI	Community Health Index
CI	Confidence interval
CIS	Continuous inpatient stay
DaSH	Data Safe Haven
ICD	International Classification of Diseases
IQR	Interquartile range
IRR	Incident rate ratio
OR	Odds ratio
SMR	Scottish Morbidity Record
SIMD	Scottish Index of Multiple Deprivation
