# Supplementary material for: Measuring multimorbidity in hospitalised patients using linked hospital episode data: comparison of two measures
Source: Int J Popul Data Sci. 2019 Jan 21;4(1):461. doi: 10.23889/ijpds.v4i1.461 (PMC7479941; doi:10.23889/ijpds.v4i1.461)
Supplement: Supplementary Appendix 1. Coding definitions (ICD-10) for conditions included in Charlson and Tonelli measures. [file ijpds-04-461-s001.pdf]

Supplementary Appendix 1. Coding definitions (ICD-10) for conditions included in Charlson and Tonelli measures

Charlson conditions (Quan 2005)

Condition	ICD-10 codes	Description	Prevalence (%)
Chronic pulmonary disease (includes asthma)	I27.8	Other specified pulmonary heart diseases	11.3
	I27.9	Pulmonary heart disease, unspecified	
	J40	Bronchitis, not specified as acute or chronic	
	J41	Simple and mucopurulent chronic bronchitis	
	J42	Unspecified chronic bronchitis	
	J43	Emphysema	
	J44	Other chronic obstructive pulmonary disease	
	J45	Asthma	
	J46	Status asthmaticus	
	J47	Bronchiectasis	
	J60	Coalworker pneumoconiosis	
	J61	Pneumoconiosis due to asbestos and other mineral fibres	
	J62	Pneumoconiosis due to dust containing silica	
	J63	Pneumoconiosis due to other inorganic dusts	
	J64	Unspecified pneumoconiosis	
	J65	Pneumoconiosis associated with tuberculosis	
	J66	Airway disease due to specific organic dust	
	J67	Hypersensitivity pneumonitis due to organic dust	
	J68.4	Chronic respiratory conditions due to chemicals, gases, fumes and vapours	
	J70.1	Chronic and other pulmonary manifestations due to radiation	
	J70.3	Chronic drug-induced interstitial lung disorders	
Diabetes without chronic complications	E10.0	Insulin-dependent diabetes mellitus-With coma	7.5
	E10.1	Insulin-dependent diabetes mellitus-With ketoacidosis	
	E10.6	Insulin-dependent diabetes mellitus-With other specified complications	
	E10.8	Insulin-dependent diabetes mellitus-With unspecified complications	
	E10.9	Insulin-dependent diabetes mellitus-Without complications	
	E11.0	Non-insulin-dependent diabetes mellitus-With coma	
	E11.1	Non-insulin-dependent diabetes mellitus-With ketoacidosis	
	E11.6	Non-insulin-dependent diabetes mellitus-With other specified complications	
	E11.8	Non-insulin-dependent diabetes mellitus-With unspecified complications	
	E11.9	Non-insulin-dependent diabetes mellitus-Without complications	
	E12.0	Malnutrition-related diabetes mellitus-With coma	
	E12.1	Malnutrition-related diabetes mellitus-With ketoacidosis	
	E12.6	Malnutrition-related diabetes mellitus-With other specified complications	
	E12.8	Malnutrition-related diabetes mellitus-With unspecified complications	

Condition	ICD-10 codes	Description	Prevalence (%)
	E12.9	Malnutrition-related diabetes mellitus-Without complications	
	E13.0	Other specified diabetes mellitus-With coma	
	E13.1	Other specified diabetes mellitus-With ketoacidosis	
	E13.6	Other specified diabetes mellitus-With other specified complications	
	E13.8	Other specified diabetes mellitus-With unspecified complications	
	E13.9	Other specified diabetes mellitus-Without complications	
	E14.0	Unspecified diabetes mellitus-With coma	
	E14.1	Unspecified diabetes mellitus-With ketoacidosis	
	E14.6	Unspecified diabetes mellitus-With other specified complications	
	E14.8	Unspecified diabetes mellitus-With unspecified complications	
	E14.9	Unspecified diabetes mellitus-Without complications	
Any malignancy, including lymphoma and leukemia, except malignant neoplasm of skin	C00–C26	Malignant neoplasms of lip, oral cavity and pharynx<br>Malignant neoplasms of digestive organs	6.9
	C30	Malignant neoplasm of nasal cavity and middle ear	
	C31	Malignant neoplasm of accessory sinuses	
	C32	Malignant neoplasm of larynx	
	C33	Malignant neoplasm of trachea	
	C34	Malignant neoplasm of bronchus and lung	
	C37	Malignant neoplasm of thymus	
	C38	Malignant neoplasm of heart, mediastinum and pleura	
	C39	Malignant neoplasm of other and ill-defined sites in the respiratory system and intrathoracic organs	
	C40	Malignant neoplasm of bone and articular cartilage of limbs	
	C41	Malignant neoplasm of bone and articular cartilage of other and unspecified sites	
	C43	Malignant melanoma of skin	
	C45–C58	Malignant neoplasms of mesothelial and soft tissue<br>Malignant neoplasm of breast<br>Malignant neoplasms of female genital organs	
	C60–C76	Malignant neoplasms of male genital organs<br>Malignant neoplasms of urinary tract<br>Malignant neoplasms of eye, brain and other parts of central nervous system<br>Malignant neoplasms of thyroid and other endocrine glands<br>Malignant neoplasm of other and ill-defined sites	
	C81	Hodgkin lymphoma	
	C82	Follicular lymphoma	
	C83	Non-follicular lymphoma	
	C84	Mature T/NK-cell lymphomas	
	C85	Other and unspecified types of non-Hodgkin lymphoma	
	C88	Malignant immunoproliferative diseases	
	C90	Multiple myeloma and malignant plasma cell neoplasms	
	C91	Lymphoid leukaemia	

Condition	ICD-10 codes	Description	Prevalence (%)
	C92	Myeloid leukaemia	
	C93	Monocytic leukaemia	
	C94	Other leukaemias of specified cell type	
	C95	Leukaemia of unspecified cell type	
	C96	Other and unspecified malignant neoplasms of lymphoid, haematopoietic and related tissue	
	C97	Malignant neoplasms of independent (primary) multiple sites	
Renal disease	I12.0	Hypertensive renal disease with renal failure	6.1
	I13.1	Hypertensive heart and renal disease with renal failure	
	N03.2	Chronic nephritic syndrome-Diffuse membranous glomerulonephritis	
	N03.3	Chronic nephritic syndrome-Diffuse mesangial proliferative glomerulonephritis	
	N03.4	Chronic nephritic syndrome-Diffuse endocapillary proliferative glomerulonephritis	
	N03.5	Chronic nephritic syndrome-Diffuse mesangiocapillary glomerulonephritis	
	N03.6	Chronic nephritic syndrome-Dense deposit disease	
	N03.7	Chronic nephritic syndrome-Diffuse crescentic glomerulonephritis	
	N05.2	Unspecified nephritic syndrome-Diffuse membranous glomerulonephritis	
	N05.3	Unspecified nephritic syndrome-Diffuse mesangial proliferative glomerulonephritis	
	N05.4	Unspecified nephritic syndrome-Diffuse endocapillary proliferative glomerulonephritis	
	N05.5	Unspecified nephritic syndrome-Diffuse mesangiocapillary glomerulonephritis	
	N05.6	Unspecified nephritic syndrome-Dense deposit disease	
	N05.7	Unspecified nephritic syndrome-Diffuse crescentic glomerulonephritis	
	N18	Chronic kidney disease	
	N19	Unspecified kidney failure	
	N25.0	Renal osteodystrophy	
	Z49.0	Preparatory care for dialysis	
	Z49.1	Extracorporeal dialysis	
	Z49.2	Other dialysis	
	Z94.0	Kidney transplant status	
	Z99.2	Dependence on renal dialysis	
Myocardial infarction	I21	Acute myocardial infarction	5.6
	I22	Subsequent myocardial infarction	
	I25.2	Old myocardial infarction	
Congestive heart failure	I09.9	Rheumatic heart disease, unspecified	3.8
	I11.0	Hypertensive heart disease with (congestive) heart failure	
	I13.0	Hypertensive heart and renal disease with (congestive) heart failure	
	I13.2	Hypertensive heart and renal disease with both (congestive) heart failure and renal failure	
	I25.5	Ischaemic cardiomyopathy	

Condition	ICD-10 codes	Description	Prevalence (%)
	I42.0	Dilated cardiomyopathy	
	I42.5	Other restrictive cardiomyopathy	
	I42.6	Alcoholic cardiomyopathy	
	I42.7	Cardiomyopathy due to drugs and other external agents	
	I42.8	Other cardiomyopathies	
	I42.9	Cardiomyopathy, unspecified	
	I43	Cardiomyopathy in diseases classified elsewhere	
	I50	Heart failure	
	P29.0	Neonatal cardiac failure	
Peripheral vascular disease	I70	Atherosclerosis	3.3
	I71	Aortic aneurysm and dissection	
	I73.1	Thromboangiitis obliterans [Buerger]	
	I73.8	Other specified peripheral vascular diseases	
	I73.9	Peripheral vascular disease, unspecified	
	I77.1	Stricture of artery	
	I79.0	Aneurysm of aorta in diseases classified elsewhere	
	I79.2	Peripheral angiopathy in diseases classified elsewhere	
	K55.1	Chronic vascular disorders of intestine	
	K55.8	Other vascular disorders of intestine	
	K55.9	Vascular disorder of intestine, unspecified	
	Z95.8	Presence of other cardiac and vascular implants and grafts	
	Z95.9	Presence of cardiac and vascular implant and graft, unspecified	
Cerebrovascular disease	G45	Transient cerebral ischaemic attacks and related syndromes	3.2
	G46	Vascular syndromes of brain in cerebrovascular diseases	
	H34.0	Transient retinal artery occlusion	
	I60	Subarachnoid haemorrhage	
	I61	Intracerebral haemorrhage	
	I62	Other nontraumatic intracranial haemorrhage	
	I63	Cerebral infarction	
	I64	Stroke, not specified as haemorrhage or infarction	
	I65	Occlusion and stenosis of precerebral arteries, not resulting in cerebral infarction	
	I66	Occlusion and stenosis of cerebral arteries, not resulting in cerebral infarction	
	I67	Other cerebrovascular diseases	
	I68	Cerebrovascular disorders in diseases classified elsewhere	
	I69	Sequelae of cerebrovascular disease	
Metastatic solid tumour	C77	Secondary and unspecified malignant neoplasm of lymph nodes	1.8
	C78	Secondary malignant neoplasm of respiratory and digestive organs	
	C79	Secondary malignant neoplasm of other and unspecified sites	
	C80	Malignant neoplasm, without specification of site	
Dementia	F00	Dementia in Alzheimer disease	1.7
	F01	Vascular dementia	

Condition	ICD-10 codes	Description	Prevalence (%)
	F02	Dementia in other diseases classified elsewhere	
	F03	Unspecified dementia	
	F05.1	Delirium superimposed on dementia	
	G30	Alzheimer disease	
	G31.1	Senile degeneration of brain, not elsewhere classified	
Rheumatic disease	M05	Seropositive rheumatoid arthritis	1.7
	M06	Other rheumatoid arthritis	
	M31.5	Giant cell arteritis with polymyalgia rheumatica	
	M32	Systemic lupus erythematosus	
	M33	Dermatopolymyositis	
	M34	Systemic sclerosis	
	M35.1	Other overlap syndromes	
	M35.3	Polymyalgia rheumatica	
	M36.0	Dermato(poly)myositis in neoplastic disease	
Mild liver disease	B18	Chronic viral hepatitis	1.4
	K70.0	Alcoholic fatty liver	
	K70.1	Alcoholic hepatitis	
	K70.2	Alcoholic fibrosis and sclerosis of liver	
	K70.3	Alcoholic cirrhosis of liver	
	K70.9	Alcoholic liver disease, unspecified	
	K71.3	Toxic liver disease with chronic persistent hepatitis	
	K71.4	Toxic liver disease with chronic lobular hepatitis	
	K71.5	Toxic liver disease with chronic active hepatitis	
	K71.7	Toxic liver disease with fibrosis and cirrhosis of liver	
	K73	Chronic hepatitis, not elsewhere classified	
	K74	Fibrosis and cirrhosis of liver	
	K76.0	Fatty (change of) liver, not elsewhere classified	
	K76.2	Central haemorrhagic necrosis of liver	
	K76.3	Infarction of liver	
	K76.4	Peliosis hepatis	
	K76.8	Other specified diseases of liver	
	K76.9	Liver disease, unspecified	
	Z94.4	Liver transplant status	
Peptic ulcer disease	K25	Gastric ulcer	0.9
	K26	Duodenal ulcer	
	K27	Peptic ulcer, site unspecified	
	K28	Gastrojejunal ulcer	
Diabetes with chronic complications	E10.2	Insulin-dependent diabetes mellitus-With renal complications	0.9
	E10.3	Insulin-dependent diabetes mellitus-With ophthalmic complications	
	E10.4	Insulin-dependent diabetes mellitus-With neurological complications	
	E10.5	Insulin-dependent diabetes mellitus-With peripheral circulatory complications	
	E10.7	Insulin-dependent diabetes mellitus-With multiple complications	
	E11.2	Non-insulin-dependent diabetes mellitus-With renal complications	
	E11.3	Non-insulin-dependent diabetes mellitus-With ophthalmic complications	

Condition	ICD-10 codes	Description	Prevalence (%)
	E11.4	Non-insulin-dependent diabetes mellitus-With neurological complications	
	E11.5	Non-insulin-dependent diabetes mellitus-With peripheral circulatory complications	
	E11.7	Non-insulin-dependent diabetes mellitus-With multiple complications	
	E12.2	Malnutrition-related diabetes mellitus-With renal complications	
	E12.3	Malnutrition-related diabetes mellitus-With ophthalmic complications	
	E12.4	Malnutrition-related diabetes mellitus-With neurological complications	
	E12.5	Malnutrition-related diabetes mellitus-With peripheral circulatory complications	
	E12.7	Malnutrition-related diabetes mellitus-With multiple complications	
	E13.2	Other specified diabetes mellitus-With renal complications	
	E13.3	Other specified diabetes mellitus-With ophthalmic complications	
	E13.4	Other specified diabetes mellitus-With neurological complications	
	E13.5	Other specified diabetes mellitus-With peripheral circulatory complications	
	E13.7	Other specified diabetes mellitus-With multiple complications	
	E14.2	Unspecified diabetes mellitus-With renal complications	
	E14.3	Unspecified diabetes mellitus-With ophthalmic complications	
	E14.4	Unspecified diabetes mellitus-With neurological complications	
	E14.5	Unspecified diabetes mellitus-With peripheral circulatory complications	
	E14.7	Unspecified diabetes mellitus-With multiple complications	
Hemiplegia or paraplegia	G04.1	Tropical spastic paraplegia	0.7
	G11.4	Hereditary spastic paraplegia	
	G80.1	Spastic diplegic cerebral palsy	
	G80.2	Spastic hemiplegic cerebral palsy	
	G81	Hemiplegia	
	G82	Paraplegia and tetraplegia	
	G83.0	Diplegia of upper limbs	
	G83.1	Monoplegia of lower limb	
	G83.2	Monoplegia of upper limb	
	G83.3	Monoplegia, unspecified	
	G83.4	Cauda equina syndrome	
	G83.9	Paralytic syndrome, unspecified	
Moderate/severe liver disease	I85.0	Oesophageal varices with bleeding	0.6
	I85.9	Oesophageal varices without bleeding	
	I86.4	Gastric varices	

Condition	ICD-10 codes	Description	Prevalence (%)
	I98.2	Oesophageal varices without bleeding in diseases classified elsewhere	
	K70.4	Alcoholic hepatic failure	
	K71.1	Toxic liver disease with hepatic necrosis	
	K72.1	Chronic hepatic failure	
	K72.9	Hepatic failure, unspecified	
	K76.5	Hepatic veno-occlusive disease	
	K76.6	Portal hypertension	
	K76.7	Hepatorenal syndrome	
AIDS/HIV	B20	Human immunodeficiency virus [HIV] disease resulting in infectious and parasitic diseases	0.0
	B21	Human immunodeficiency virus [HIV] disease resulting in malignant neoplasms	
	B22	Human immunodeficiency virus [HIV] disease resulting in other specified diseases	
	B24	Unspecified human immunodeficiency virus [HIV] disease	

Tonelli conditions (Tonelli 2015)

Morbidity	ICD-10 codes	Description	Prevalence (%)
Hypertension	I10<br>I11<br>I12<br>I13<br>I15	Essential (primary) hypertension<br>Hypertensive heart disease<br>Hypertensive renal disease<br>Hypertensive heart and renal disease<br>Secondary hypertension	19.0
Diabetes	E10<br>E11<br>E12<br>E13<br>E14	Insulin-dependent diabetes mellitus<br>Non-insulin-dependent diabetes mellitus<br>Malnutrition-related diabetes mellitus<br>Other specified diabetes mellitus<br>Unspecified diabetes mellitus	8.4
Chronic kidney disease	N00-N23	Glomerular diseases (N00-N08)<br>Renal tubulo-interstitial diseases (N10-N16)<br>Renal failure (N17-N19)<br>Urolithiasis (N20-N23)	8.2
Asthma	J45	Asthma	6.7
Atrial fibrillation and flutter	I48	Atrial fibrillation and flutter	6.1
Chronic pain	F45.4<br>M08.1<br>M25.5<br>M43.2<br>M43.3<br>M43.4<br>M43.5<br>M43.6<br>M45<br>M46.1<br>M46.3<br>M46.4<br>M46.9<br>M47<br>M48.0<br>M48.1<br>M48.8<br>M48.9<br>M50.8<br>M50.9<br>M51<br>M53.1<br>M53.2<br>M53.3<br>M53.8<br>M53.9<br>M54<br>M60.8<br>M60.9<br>M63.3<br>M79.0<br>M79.1<br>M79.2	Persistent somatoform pain disorder<br>Juvenile ankylosing spondylitis<br>Pain in joint<br>Other fusion of spine<br>Recurrent atlantoaxial subluxation with myelopathy<br>Other recurrent atlantoaxial subluxation<br>Other recurrent vertebral subluxation<br>Torticollis<br>Ankylosing spondylitis<br>Sacroiliitis, not elsewhere classified<br>Infection of intervertebral disc (pyogenic)<br>Discitis, unspecified<br>Inflammatory spondylopathy, unspecified<br>Spondylosis<br>Spinal stenosis<br>Ankylosing hyperostosis [Forestier]<br>Other specified spondylopathies<br>Spondylopathy, unspecified<br>Other cervical disc disorders<br>Cervical disc disorder, unspecified<br>Other intervertebral disc disorders<br>Cervicobrachial syndrome<br>Spinal instabilities<br>Sacrococcygeal disorders, not elsewhere classified<br>Other specified dorsopathies<br>Dorsopathy, unspecified<br>Dorsalgia<br>Other myositis<br>Myositis, unspecified<br>Myositis in sarcoidosis<br>Rheumatism, unspecified<br>Myalgia<br>Neuralgia and neuritis, unspecified	6.1

Morbidity	ICD-10 codes	Description	Prevalence (%)
	M79.6	Pain in limb	
	M79.7	Fibromyalgia	
	M96.1	Postlaminectomy syndrome, not elsewhere classified	
Chronic pulmonary disease (excludes asthma)	I27.8	Other specified pulmonary heart diseases	5.9
	I27.9	Pulmonary heart disease, unspecified	
	J40	Bronchitis, not specified as acute or chronic	
	J41	Simple and mucopurulent chronic bronchitis	
	J42	Unspecified chronic bronchitis	
	J43	Emphysema	
	J44	Other chronic obstructive pulmonary disease	
	J46	Status asthmaticus	
	J47	Bronchiectasis	
	J60	Coalworker pneumoconiosis	
	J61	Pneumoconiosis due to asbestos and other mineral fibres	
	J62	Pneumoconiosis due to dust containing silica	
	J63	Pneumoconiosis due to other inorganic dusts	
	J64	Unspecified pneumoconiosis	
	J65	Pneumoconiosis associated with tuberculosis	
	J66	Airway disease due to specific organic dust	
	J67	Hypersensitivity pneumonitis due to organic dust	
	J68.4	Chronic respiratory conditions due to chemicals, gases, fumes and vapours	
	J70.1	Chronic and other pulmonary manifestations due to radiation	
	J70.3	Chronic drug-induced interstitial lung disorders	
Hypothyroidism	E00	Congenital iodine-deficiency syndrome	4.5
	E01	Iodine-deficiency-related thyroid disorders and allied conditions	
	E02	Subclinical iodine-deficiency hypothyroidism	
	E03	Other hypothyroidism	
	E89.0	Postprocedural hypothyroidism	
Cancer, non-metastatic (breast, cervical, colorectal, lung, prostate)	C18	Malignant neoplasm of colon	4.2
	C19	Malignant neoplasm of rectosigmoid junction	
	C20	Malignant neoplasm of rectum	
	C21	Malignant neoplasm of anus and anal canal	
	C33	Malignant neoplasm of trachea	
	C34	Malignant neoplasm of bronchus and lung	
	C38.4	Pleura	
	C45.0	Mesothelioma of pleura	
	C46.7	Kaposi sarcoma of other sites	
	C50	Malignant neoplasm of breast	
	C53	Malignant neoplasm of cervix uteri	
	C61	Malignant neoplasm of prostate	
	D01.0	Carcinoma in situ of Colon	
	D01.1	Carcinoma in situ of Rectosigmoid junction	
	D01.2	Carcinoma in situ of Rectum	
	D01.3	Carcinoma in situ of Anus and anal canal	
	D02.2	Carcinoma in situ of Bronchus and lung	
	D05	Carcinoma in situ of breast	

Morbidity	ICD-10 codes	Description	Prevalence (%)
	D06	Carcinoma in situ of cervix uteri	
	D07.5	Carcinoma in situ of Prostate	
Alcohol misuse	E52	Niacin deficiency	4.1
	F10	Mental and behavioural disorders due to use of alcohol	
	G62.1	Alcoholic polyneuropathy	
	I42.6	Alcoholic cardiomyopathy	
	K29.2	Alcoholic gastritis	
	K70.0	Alcoholic fatty liver	
	K70.3	Alcoholic cirrhosis of liver	
	K70.9	Alcoholic liver disease, unspecified	
	T51	Toxic effect of alcohol	
	Z50.2	Alcohol rehabilitation	
	Z71.4	Alcohol abuse counselling and surveillance	
	Z72.1	Alcohol use	
Chronic heart failure	I09.9	Rheumatic heart disease, unspecified	3.8
	I25.5	Ischaemic cardiomyopathy	
	I42.0	Dilated cardiomyopathy	
	I42.5	Other restrictive cardiomyopathy	
	I42.6	Alcoholic cardiomyopathy	
	I42.7	Cardiomyopathy due to drugs and other external agents	
	I42.8	Other cardiomyopathies	
	I42.9	Cardiomyopathy, unspecified	
	I43	Cardiomyopathy in diseases classified elsewhere	
	I50	Heart failure	
Severe constipation	K55.8	Other vascular disorders of intestine	3.3
	K56.0	Paralytic ileus	
	K56.4	Other impaction of intestine	
	K56.7	Ileus, unspecified	
	K59.0	Constipation	
	K63.1	Perforation of intestine (nontraumatic)	
	K63.4	Enteroptosis	
	K63.8	Other specified diseases of intestine	
	K92.8	Other specified diseases of digestive system	
Myocardial infarction	I21	Acute myocardial infarction	2.3
	I22	Subsequent myocardial infarction	
Depression	F20.4	Post-schizophrenic depression	2.3
	F31.3	Bipolar affective disorder, current episode mild or moderate depression	
	F31.4	Bipolar affective disorder, current episode severe depression without psychotic symptoms	
	F31.5	Bipolar affective disorder, current episode severe depression with psychotic symptoms	
	F32	Depressive episode	
	F33	Recurrent depressive disorder	
	F34.1	Dysthymia	
	F41.2	Mixed anxiety and depressive disorder	
	F43.2	Adjustment disorders	

Morbidity	ICD-10 codes	Description	Prevalence (%)
Stroke or transient ischaemic attack	G45.0	Vertebro-basilar artery syndrome	2.0
	G45.1	Carotid artery syndrome (hemispheric)	
	G45.2	Multiple and bilateral precerebral artery syndromes	
	G45.3	Amaurosis fugax	
	G45.8	Other transient cerebral ischaemic attacks and related syndromes	
	G45.9	Transient cerebral ischaemic attack, unspecified	
	H34.1	Central retinal artery occlusion	
	I60	Subarachnoid haemorrhage	
	I61	Intracerebral haemorrhage	
	I63	Cerebral infarction	
	I64	Stroke, not specified as haemorrhage or infarction	
Cancer, metastatic	C77	Secondary and unspecified malignant neoplasm of lymph nodes	1.8
	C78	Secondary malignant neoplasm of respiratory and digestive organs	
	C79	Secondary malignant neoplasm of other and unspecified sites	
	C80	Malignant neoplasm, without specification of site	
Dementia	F00	Dementia in Alzheimer disease	1.7
	F01	Vascular dementia	
	F02	Dementia in other diseases classified elsewhere	
	F03	Unspecified dementia	
	F05.1	Delirium superimposed on dementia	
	G30	Alzheimer disease	
	G31.1	Senile degeneration of brain, not elsewhere classified	
Rheumatoid arthritis	M05	Seropositive rheumatoid arthritis	1.7
	M06	Other rheumatoid arthritis	
	M31.5	Giant cell arteritis with polymyalgia rheumatica	
	M32	Systemic lupus erythematosus	
	M33	Dermatopolymyositis	
	M34	Systemic sclerosis	
	M35.1	Other overlap syndromes	
	M35.3	Polymyalgia rheumatica	
	M36.0	Dermato(poly)myositis in neoplastic disease	
Epilepsy	G40	Epilepsy	1.4
	G41	Status epilepticus	
Inflammatory bowel disease	K50	Crohn disease [regional enteritis]	1.4
	K51	Ulcerative colitis	
Cirrhosis and hepatic decompensation	K70.3	Alcoholic cirrhosis of liver	1.1
	K74.3	Primary biliary cirrhosis	
	K74.4	Secondary biliary cirrhosis	
	K74.5	Biliary cirrhosis, unspecified	
	K74.6	Other and unspecified cirrhosis of liver	
	I85.0	Oesophageal varices with bleeding	
	I85.9	Oesophageal varices without bleeding	
	I98.2	Oesophageal varices without bleeding in diseases classified elsewhere	
	I98.3	Oesophageal varices with bleeding in diseases classified elsewhere	
	K65.0	Acute peritonitis	

Morbidity	ICD-10 codes	Description	Prevalence (%)
	K65.8	Other peritonitis	
	K65.9	Peritonitis, unspecified	
	K67.0	Chlamydial peritonitis	
	K67.1	Gonococcal peritonitis	
	K67.2	Syphilitic peritonitis	
	K67.3	Tuberculous peritonitis	
	K67.8	Other disorders of peritoneum in infectious diseases classified elsewhere	
	K76.7	Hepatorenal syndrome	
	K93.0	Tuberculous disorders of intestines, peritoneum and mesenteric glands	
	R18	Ascites	
Cancer, lymphoma	C81	Hodgkin lymphoma	0.6
	C82	Follicular lymphoma	
	C83	Non-follicular lymphoma	
	C84	Mature T/NK-cell lymphomas	
	C85	Other and unspecified types of non-Hodgkin lymphoma	
	C88	Malignant immunoproliferative diseases	
	C90.0	Multiple myeloma	
	C90.2	Extramedullary plasmacytoma	
	C96	Other leukaemias of specified cell type	
Peptic ulcer disease without haemorrhage	K25.7	Gastric ulcer-Chronic without haemorrhage or perforation	0.6
	K25.9	Gastric ulcer-Unspecified as acute or chronic, without haemorrhage or perforation	
	K26.7	Duodenal ulcer-Chronic without haemorrhage or perforation	
	K26.9	Duodenal ulcer-Unspecified as acute or chronic, without haemorrhage or perforation	
	K27.7	Peptic ulcer, site unspecified- Chronic without haemorrhage or perforation	
	K27.9	Peptic ulcer, site unspecified- Unspecified as acute or chronic, without haemorrhage or perforation	
	K28.7	Gastrojejunal ulcer- Chronic without haemorrhage or perforation	
	K28.9	Gastrojejunal ulcer- Unspecified as acute or chronic, without haemorrhage or perforation	
Irritable bowel syndrome	K58	Irritable bowel syndrome	0.5
Multiple sclerosis	G35	Multiple sclerosis	0.5
	G36	Other acute disseminated demyelination	
	G37	Other demyelinating diseases of central nervous system	
	H46	Optic neuritis	
Parkinson’s disease	G20	Parkinson disease	0.5
	G21	Secondary parkinsonism	
	G22	Parkinsonism in diseases classified elsewhere	
Schizophrenia	F20	Schizophrenia	0.3
	F21	Schizotypal disorder	
	F23.2	Acute schizophrenia-like psychotic disorder	
	F25	Schizoaffective disorders	

Morbidity	ICD-10 codes	Description	Prevalence (%)
Psoriasis	L40.0	Psoriasis vulgaris	0.2
	L40.1	Generalized pustular psoriasis	
	L40.2	Acrodermatitis continua	
	L40.3	Pustulosis palmaris et plantaris	
	L40.4	Guttate psoriasis	
	L40.8	Other psoriasis	
	L40.9	Psoriasis, unspecified	
Peripheral vascular disease	I70.2	Atherosclerosis of arteries of extremities	0.1
Chronic viral hepatitis B	B16	Acute hepatitis B	0.0
	B18.0	Chronic viral hepatitis B with delta-agent	
	B18.1	Chronic viral hepatitis B without delta-agent	

Notes: Minor changes to the Tonelli et al. (2015) published algorithms were made. The algorithms developed by Tonelli required a combination of hospitalization, claims and outpatient codes over varying time periods. We modified this to at least one hospitalisation in the five years prior to index for all morbidities. For identifying chronic kidney disease, Tonelli supplemented the algorithm using lab data, however we only used administrative data. The codes for “peptic ulcer disease” referred to “peptic ulcer disease without haemorrhage or perforation”, therefore we amended the morbidity description. As specific codes for atrial fibrillation were only implemented in Scotland from April 2016 (ICD-10 version 2016), we included “atrial fibrillation and flutter” (ICD-10 code I48). No entry in ICD-10 was identified for: C46.71 (replaced with C46.7); M25.50, M25.51, M25.55, M25.57 (replaced with M25.5); K63.81 and K63.88 (replaced with K63.8), K92.80 and K92.88 (replaced with K92.8).
