# Supplementary material for: Measuring multimorbidity in hospitalised patients using linked hospital episode data: comparison of two measures
Source: Int J Popul Data Sci. 2019 Jan 21;4(1):461. doi: 10.23889/ijpds.v4i1.461 (PMC7479941; doi:10.23889/ijpds.v4i1.461)
Supplement: Supplementary Appendix 2. Characteristics of patients with missing CHI numbers, SIMD quintile or UR category. [file ijpds-04-461-s002.pdf]

Supplementary Appendix 2. Characteristics of patients with missing CHI numbers, SIMD quintile or UR category

Admissions with missing CHI numbers, adults with an inpatient stay 2014

Records with missing CHI numbers had a higher proportion of males and younger age than our final population.

Age-sex distribution of admissions with missing CHI numbers

Age	Female	Male	Total
18-20	8	17	**25**
21-30	33	111	**144**
31-40	17	86	**103**
41-50	29	88	**117**
51-60	18	84	**102**
61-70	30	47	**77**
71-80	33	29	**62**
≥81	19	13	**32**
**Total**	**187**	**475**	**662**

Patients with missing SIMD quintile and UR category

Characteristic	SIMD missing		UR missing	
	n	%	n	%
Total	***314***		***576***	
**Males, n (%)**	155	(49.4)	258	(44.8)
**Age, median (IQR)**	47	(31-62)	43	(29-60
**Admission type, n (%)**				
**Routine**	82	(26.1)	156	(27.1)
**Emergency**	232	(73.9)	420	(72.9)
