# Supplementary material for: Measuring multimorbidity in hospitalised patients using linked hospital episode data: comparison of two measures
Source: Int J Popul Data Sci. 2019 Jan 21;4(1):461. doi: 10.23889/ijpds.v4i1.461 (PMC7479941; doi:10.23889/ijpds.v4i1.461)
Supplement: Supplementary Appendix 3. Tonelli et al. measure observations. [file ijpds-04-461-s003.pdf]

Supplementary Appendix 3. Tonelli et al. measure observations

As Tonelli et al. (2015) did not identify appropriate algorithms for all 40 conditions included in the Barnett et al. (2012) measure, the final list included 30 conditions, equating to 27 of the 40 (plus two additional cancer algorithms plus peptic ulcer disease). The 13 conditions not included in the Tonelli measure were:

- Anorexia or bulimia
- Anxiety disorders
- Blindness
- Bronchiectasis
- Chronic sinusitis
- Diverticulosis
- Treated dyspepsia
- Glaucoma
- Hearing loss
- Learning disability
- Migraine
- Non-alcohol drug misuse
- Prostate disorders

In addition, there were also some differences in the specific conditions included:

**Barnett condition**	**Tonelli condition**
Chronic liver disease	Cirrhosis and hepatic decompensation
Coronary heart disease	Myocardial infarction
New diagnosis of cancer in last five years	Cancer, lymphoma
	Cancer, metastatic
	Cancer, non-metastatic (breast, cervical, colorectal, lung, prostate)
Psoriasis or eczema	Psoriasis
Rheumatoid arthritis, other inflammatory polyarthropathies & systematic connective tissue disorders	Rheumatoid arthritis
Schizophrenia (and related non-organic psychosis) or bipolar disorder	Schizophrenia
Thyroid disorders	Hypothyroidism
Viral Hepatitis	Chronic viral hepatitis B
Not included in Barnett measure	Peptic ulcer disease
